# Fibrates Affect Levels of Phosphorylated p38 in Intestinal Cells in a Differentiation-Dependent Manner

**DOI:** 10.3390/ijms24097695

**Published:** 2023-04-22

**Authors:** Katerina Cizkova, Zdenek Tauber

**Affiliations:** Department of Histology and Embryology, Faculty of Medicine and Dentistry, Palacky University, 779 00 Olomouc, Czech Republic

**Keywords:** epoxyeicosatrienoic acids, peroxisome proliferator-activated receptor α, colorectal carcinoma, fibrates, p38 MAPK

## Abstract

Fibrates are widely used hypolipidaemic agents that act as ligands of the peroxisome proliferator-activated receptor α (PPARα). p38 is a protein kinase that is mainly activated by environmental and genotoxic stress. We investigated the effect of the PPARα activators fenofibrate and WY-14643 and the PPARα inhibitor GW6471 on the levels of activated p38 (p-p38) in the colorectal cancer cell lines HT-29 and Caco2 in relation to their differentiation status. Fibrates increased p-p38 in undifferentiated HT-29 cells, whereas in other cases p-p38 expression was decreased. HT-29 cells showed p-p38 predominantly in the cytoplasm, whereas Caco2 cells showed higher nuclear positivity. The effect of fibrates may depend on the differentiation status of the cell, as differentiated HT-29 and undifferentiated Caco2 cells share similar characteristics in terms of villin, CYP2J2, and soluble epoxide hydrolase (sEH) expression. In human colorectal carcinoma, higher levels of p-p38 were detected in the cytoplasm, whereas in normal colonic surface epithelium, p-p38 showed nuclear positivity. The decrease in p-p38 positivity was associated with a decrease in sEH, consistent with in vitro results. In conclusion, fibrates affect the level of p-p38, but its exact role in the process of carcinogenesis remains unclear and further research is needed in this area.

## 1. Introduction

Fibrates, such as fenofibrate, bezafibrate, clofibrate, and gemfibrozil, are widely used hypolipidaemic agents. They are generally well-tolerated drugs with a good safety profile [1,2,3]. These molecules are known as peroxisome proliferator-activated receptor α (PPARα) ligands. PPARα is a ligand-dependent transcription factor that heterodimerises with the retinoid X receptor after ligand binding. The complex binds to peroxisome proliferator response elements (PPREs) and regulates the expression of a number of target genes [4], including genes involved in energy metabolism, oxidative stress, immune response, xenobiotic metabolism, cell proliferation, differentiation, and carcinogenesis [4,5,6,7,8]. Other synthetic compounds that activate PPARα, such as WY-14643 (or pyrinixic acid), are not approved for clinical use. However, they are often used for research purposes [1]. Besides fibrates, PPARα is also activated by phthalates, pesticides, etc. [5,6].

In addition to exogenous ligands, PPARα is also activated by endogenous molecules such as dietary fatty acids, including arachidonic acid and its metabolites [9,10,11]. Arachidonic acid is converted by CYP epoxygenases (CYP2C and CYP2J subfamilies) to four regioisomers of epoxyeicosatrienoic acids (EETs). EETs are generally cytoprotective, stimulating cell proliferation, and inhibiting apoptosis [12,13]. EETs are rapidly hydrolysed to less active dihydroxyeicosatrienoic acids (DHETs) by soluble epoxide hydrolase (sEH) or alternatively incorporated into membrane phospholipids [14]. Our previous study showed that fibrates regulate the expression levels of CYP epoxygenases as well as sEH in a dose-dependent manner, and these changes are accompanied by changes in the cell proliferation response [15]. EETs and DHETs activate PPARα, and PPARα in turn regulates the expression of EETs- and DHETs-generating enzymes, resulting in the feedback mechanism [11,16]. In addition to ligand binding, the transcriptional activity of PPARα is regulated by phosphorylation by mitogen-activated protein kinases (ERK and p38-MAPK), protein kinase A and C (PKA, PKC), AMP kinase (AMPK) and glycogen synthase kinase-3 (GSK3) in a ligand-dependent or -independent manner [17].

The intestinal epithelium is one of the most rapidly renewed tissues in the organism, with a turnover of approximately 5 days [18]. Colorectal cancer is the third-most common cancer in terms of incidence, but the second-most common in terms of mortality [19]. Fenofibrate shows promising effects on colorectal cancer in mouse models. Activation of PPARα by fenofibrate protects human PPARα transgenic mice from chemically induced colorectal cancer [20]. Furthermore, fenofibrate inhibits tumour growth and DNMT1 activity in a xenograft model of HCT116 cell-bearing nude mice. The authors of the study propose fenofibrate as an epigenetic therapy for colorectal carcinoma [21]. However, a previous meta-analysis of 17 randomised control trials did not show that the use of fibrates significantly affected the incidence of colorectal cancer or cancer-related deaths [22]. A recent review of the role of hypolipidaemic treatment in the prevention of second primary cancers in colorectal cancer survivors showed that fibrates did not have a significant chemoprotective effect against cancer. However, the authors note that the subgroup of patients treated with fibrates in their study was too small [23].

p38 is mainly activated by environmental and genotoxic stress and is known as a stress-activated protein kinase. The four p38 MAPKs, namely p38α, p38β, p38γ, and p38δ, are encoded by different genes. p38α and p38β are ubiquitously expressed, but the expression of p38β is much lower than that of p38α. The expression patterns of p38γ and p38δ are more restricted. p38 activation occurs through dual phosphorylation of Thr and Tyr. p38 regulates multiple cellular functions including cell proliferation, differentiation, stress response, apoptosis, cell migration, and survival. The exact role of p38 in carcinogenesis is not fully understood. On the one hand, it acts as a tumour suppressor by promoting cell cycle arrest, cell differentiation, senescence, and apoptosis. On the other hand, p38 promotes cancer by increasing survival, migration, or resistance to stress and chemotherapeutic agents in tumour cells. The role of p38 depends not only on the type of stimulus, but also in a cell type-specific manner and on the level of activation, as strong and sustained p38 activation has been linked to apoptosis, senescence, and terminal cell differentiation, whereas low p38 activation has a cell survival effect [24,25,26]. p38 is activated during intestinal cell differentiation [27]. p38 signalling has been proposed as a critical node in cancer and its therapy, but results are conflicting. In colorectal cancer, commonly used chemotherapeutic drugs such as 5-fluorouracil, oxaliplatin, or irinotecan activate p38 with consequences ranging from cell death to cell survival and resistance [28].

There is limited information on the effect of PPARα ligands on p38. Fenofibrate has been shown to upregulate phosphorylated p38 (p-p38) in B16-100 melanoma cells, thereby inhibiting melanin synthesis [29]. Increased p-p38 was also found in mouse brain endothelial cells after WY-14643 treatment [30]. In contrast, fenofibrate has been described to inhibit p-p38 in renal proximal tubular cells, protecting them from cisplatin-induced cytotoxicity [31]. In addition, endogenous PPARα ligands EETs could also affect the phosphorylation of p38 [32,33,34,35,36]. In terms of intestinal pathology, inhibition of p38 can effectively suppress the expression of inflammatory mediators in ulcerative colitis and Crohn’s disease [37]. In mouse models of these diseases, fenofibrate protects the intestine from colitis-induced permeability [38] and may also have a potential therapeutic role in Crohn’s disease [39].

The aim of this study was to investigate the effect of the PPARα activators fenofibrate and WY-14643 as well as the PPARα inhibitor GW6471 on the levels of p-p38 (dually phosphorylated at Thr180 and Typ182). Two colorectal carcinoma cell lines, HT-29 and Caco2, were used as models. We also investigated p-p38, CYP2J2 and sEH in human grade 2 colorectal carcinoma tissues and adjacent normal tissues.

## 2. Results

### 2.1. Effect of Fenofibrate, WY-14643 and GW6471 Treatment on p-p38 in Undifferentiated and Differentiated HT-29 and Caco2 Cells

**Figure 1:**
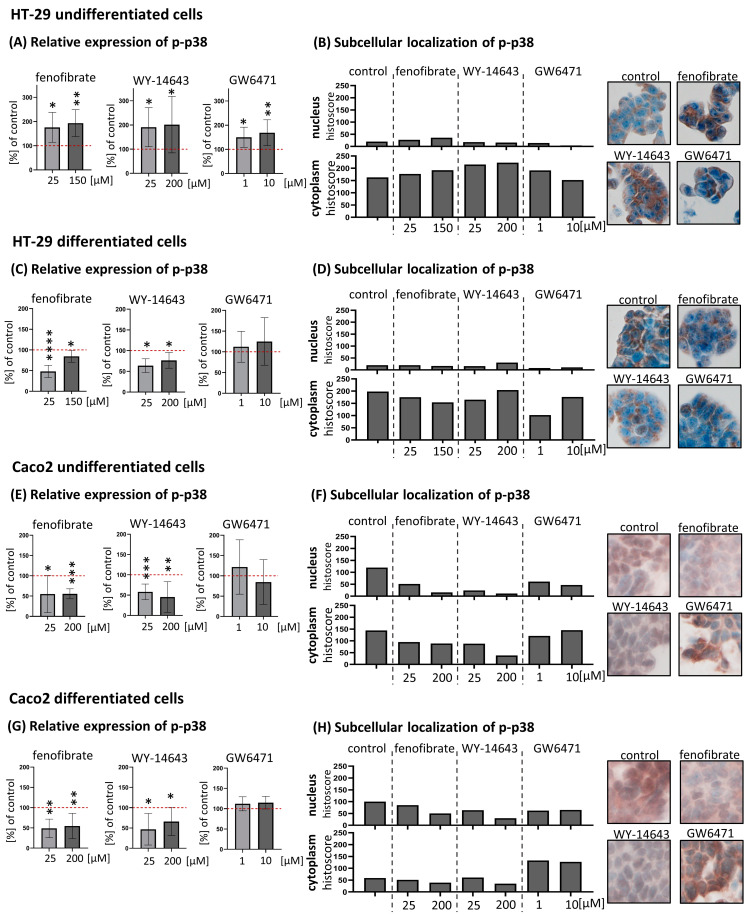
Changes in expression levels of phosphorylated p38 (p-p38) after treatment with fenofibrate, WY-14643, and GW6471. Parts (**A**,**C**,**E**,**G**) Relative expression of p-p38 compared to control was measured by In-Cell ELISA. Results are expressed as mean ± SD (*n* = 8). The red dotted lines represent control cells (100%). Statistically significant results compared to control cells are marked with (*): * *p* ≤ 0.05, ** *p* ≤ 0.01, *** *p* ≤ 0.001, **** *p* ≤ 0.0001. Parts (**B**,**D**,**F**,**H**) Subcellular localisation of p-p38 after fenofibrate, WY-14643 and GW6471 treatment obtained by immunocytochemical staining. p-p38 was evaluated separately in the nucleus and cytoplasm of the cells. The results are presented as histoscores. Cells were counted in 5 different fields of vision (magnification ×200). Microphotographs show control cells and cells treated with 150 μM (HT-29) or 200 μM (Caco2) fenofibrate, 200 μM WY-14643 and 10 μM GW6471. All images are at the same magnification (200×).

Changes in p-p38 expression were assessed by ICE (see Figure 1, parts A). Surprisingly, undifferentiated HT-29 showed a different response to PPARα ligands than the other cells tested. In undifferentiated HT-29 cells, we observed a significant increase in p-p38 expression after treatment with all three compounds tested. Fenofibrate at concentrations of 25 μM and 150 μM increased p-p38 expression to 175.80 ± 62.02 (*p* = 0.0106) and 193.60 ± 55.34 (*p* = 0.0020) % of control, respectively. WY-14643 at concentrations of 25 μM and 200 μM increased p-p38 expression to 190.50 ± 80.48 (*p* = 0.0155) and 201.30 ± 116.50 (*p* = 0.0434) % of control, respectively. The PPARα inhibitor GW6417 at concentrations of 1 μM and 10 μM increased p-p38 expression to 149.80 ± 41.85 (*p* = 0.0120) and 168.70 ± 53.33 (*p* = 0.0082) % of control, respectively.

In contrast to undifferentiated HT-29 cells, we observed a significant decrease of p-p38 in sodium butyrate-differentiated HT-29 cells after treatment with PPARα activators. Fenofibrate at concentrations of 25 μM and 150 μM decreased p-p38 expression to 48.00 ± 14.72 (*p* < 0.0001) and 84.63 ± 14.32 (*p* = 0.0466) % of control, respectively. WY-14643 at 25 μM and 200 μM reduced p38 expression to 63.80 ± 16.57 (*p* = 0.0222) and 76.44 ± 19.01 (*p* = 0.0286) % of control, respectively. Treatment with 1 μM and 10 μM GW6471 resulted in a slight non-significant increase in p-p38 expression to 112.40 ± 37.37 (*p* = 0.4545) and 125.00 ± 57.42% (*p* = 0.2577) of control, respectively.

In undifferentiated Caco2 cells, we observed a significant decrease in p-p38 after treatment with PPARα activators. Fenofibrate at concentrations of 25 μM and 200 μM decreased p-p38 expression to 55.07 ± 45.82 (*p* = 0.0127) and 55.66 ± 12.29 (*p* = 0.0003) % of control, respectively. WY-14643 at concentrations of 25 μM and 200 μM decreased p-p38 expression to 57.88 ± 19.30 (*p* = 0.0005) and 45.30 ± 37.85 (*p* = 0.0087) % of control, respectively. Treatment with 1 μM and 10 μM GW6471 resulted in non-significant changes in p-p38 expression compared to control: 121.70 ± 67.05 (*p* = 0.3591) and 84.61 ± 55.07% (*p* = 0.4551), respectively.

In spontaneously differentiated Caco2 cells, we observed a significant decrease in p-p38 after treatment with PPARα activators. Fenofibrate at concentrations of 25 μM and 200 μM decreased p-p38 expression to 48.73 ± 22.97 (*p* = 0.0010) and 54.63 ± 31.45 (*p* = 0.0025) % of control, respectively. WY-14643 at concentrations of 25 μM and 200 μM decreased p38 expression to 46.82 ± 38.97 (*p* = 0.0380) and 65.89 ± 34.83 (*p* = 0.0416) % of control, respectively. Treatment with 1 μM and 10 μM GW6471 resulted in a slight, non-significant increase in p38 expression to 112.10 ± 17.33 (*p* = 0.1143) and 114.90 ± 15.58% (*p* = 0.1526) of control, respectively.

### 2.2. Evaluation of Subcellular Localisation of p-p38 after Treatment with Fenofibrate, WY-14643 and GW6471 in Undifferentiated and Differentiated HT-29 and Caco2 Cells

Immunocytochemical staining of p-p38 was performed to assess its subcellular localisation after PPARα ligands (see Figure 1, pats B). In HT-29 cells, we detected p-p38 expression predominantly in the cytoplasm in both undifferentiated and differentiated HT-29 cells. In control cells, nuclear positivity of p-p38 was detected in 14.8% of undifferentiated cells, while cytoplasmic positivity was detected in 88.4%. Fibrate generally increased cytoplasmic level of p-p38. Moreover, fenofibrate increased nuclear level. In differentiated HT-29 cells, we detected 15.5% of cells with nuclear positivity and 91.4% of cells with cytoplasmic positivity. Contrary to undifferentiated cells, fibrates as well as GW6471 decreased cytoplasmic levels of p-p38. Nuclear and cytoplasmic positivity was also observed in Caco2 cells. Nuclear levels of p-p38 were higher than in HT-29. In undifferentiated Caco2, we detected 78.6% of cells with nuclear positivity and 92.1% of cells with positive cytoplasm. In differentiated Caco2, we detected 68.3% of cells with nuclear positivity and 52.8% of cells with positive cytoplasm. In Caco2, fibrates generally decreased both cytoplasmic and nuclear levels of p-p38.

### 2.3. Differences between HT-29 and Caco-2 Cells

**Figure 2:**
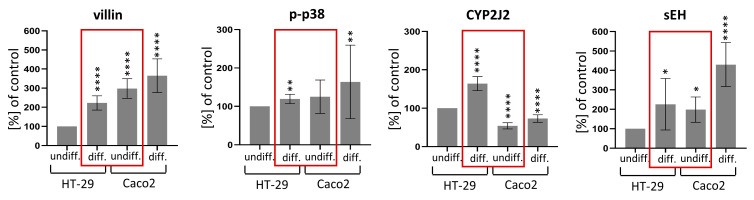
Comparison of undifferentiated and differentiated HT-29 and Caco2 cells. The relative expression of villin, p-p38, CYP2J2 and sEH was measured by in-cell ELISA. Results are presented as mean ± SD (*n* = 8). The expression of the proteins of interest in HT-29 cells was used as a control (100%). Statistically significant results compared to control cells are indicated with (*): * *p* ≤ 0.05, ** *p* ≤ 0.01, **** *p* ≤ 0.0001. Note comparable levels of p-p38 and sEH, increased levels of villin and decreased levels of CYP2J2 in undifferentiated Caco2 cells compared to differentiated HT-29, suggesting that undifferentiated Caco2 are more similar to differentiated HT-29 than undifferentiated HT-29 (red rectangles).

Because of the opposite response in p-p38 levels of undifferentiated and differentiated HT-29, undifferentiated Caco2 and differentiated Caco2 to PPARα ligands treatment, we examined the differentiation status of HT-29 and Caco2 cells (see Figure 2). Villin was used as a marker of differentiation. The expression of CYP2J2, sEH and p-p38 itself were also examined. Using undifferentiated HT-29 cells as a control (100%), we detected significantly increased villin levels in differentiated HT-29, undifferentiated Caco2, and differentiated Caco2 cells (one-sample *t*-test, *p* < 0.0001 for all cases). We also found that differentiated HT-29 cells and undifferentiated Caco2 cells differed significantly in villin expression (unpaired *t*-test, *p* = 0.0033).

CYP2J2 expression is lower in undifferentiated Caco2 cells compared to undifferentiated HT-29 cells (*p* < 0.0001). In contrast, sEH expression is higher in undifferentiated Caco2 than in undifferentiated HT-29 cells (*p* = 0.0279). Both enzymes are increased in differentiated cells compared to undifferentiated cells in both cell lines. The increase of p-p38 in undifferentiated Caco2 compared to undifferentiated HT-29 cells approached significance with *p* = 0.0990. p-p38 is also increased in differentiated compared to undifferentiated cells in both cell lines.

### 2.4. Immunohistochemical Staining of p-p38, CYP2J2, and sEH in Grade 2 Colorectal Carcinoma and Adjacent Normal Tissue Samples

**Figure 3:**
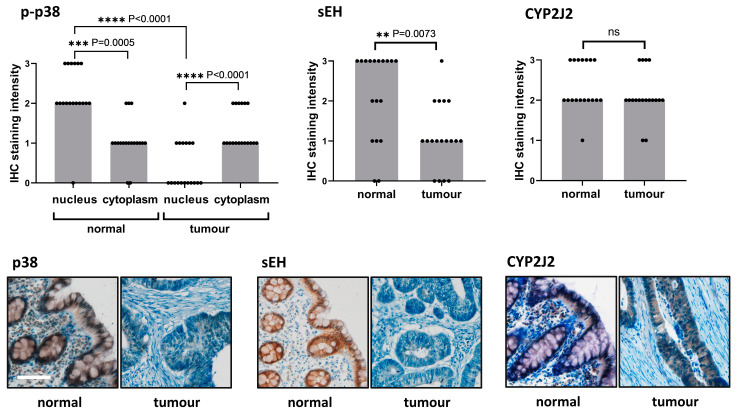
Immunohistochemical staining for p-p38, CYP2J2, and sEH in normal and colorectal carcinoma samples. Representative micrographs of grade 2 tumours and corresponding normal tissue samples were obtained from the same patient (male, 55 years old). Magnification ×100, white line represents 100 μm. Graphs show immunohistochemical staining intensities (*n* = 18): columns show medians, dots show individual samples. Immunostaining intensities were scored semiquantitatively as: 0 negative staining, 1 weak staining, 2 moderate staining, 3 strong staining. Immunostaining for p-p38 was scored separately for nucleus and cytoplasm, CYP2J2 and sEH were scored in cytoplasm. Comparison of staining intensity between normal and carcinoma tissues was performed by the Wilcoxon signed rank test at a significance level of *p* ≤ 0.05. Statistically significant results are marked with *, *p*-values are given directly in the graphs, ns—nonsignificant result.

We found significant differences between the immunostaining intensity of p-p38 in the nucleus and cytoplasm in both normal adjacent colonic surface epithelium and in colorectal carcinomas (see Figure 3). In normal epithelium, p-p38 expression was higher in the nucleus than in the cytoplasm (median 2 vs. median 1, *p* < 0.0001). In contrast, in colorectal carcinoma, p-p38 expression was higher in the cytoplasm than in the nucleus (median 0 vs. median 1, *p* < 0.0001). Nuclear positivity of p-p38 was significantly higher in normal epithelium than in carcinoma (median 2 vs. median 0, *p* < 0.0001) whereas cytoplasmic immunostaining was stronger in carcinoma than in normal epithelium (*p* = 0.0116). The expression pattern of p-p38 in tumours was not affected by sex, tumour size, or lymph node infiltration.

Immunostaining of sEH was significantly decreased in carcinoma compared to normal epithelium (median 1 vs. median 3, *p* = 0.0015). In contrast to p38 and sEH, CYP2J2 immunostaining intensities were comparable in normal adjacent tissue and colorectal carcinoma (*p* = 0.3984).

## 3. Discussion

We investigated the effect of the PPARα activators fenofibrate and WY-14643 and the PPARα inhibitor GW6471 on the levels of activated p38 (p-p38) in the colorectal cancer cell lines HT-29 and Caco2 cells in relation to their differentiation status. We also investigated p-p38, CYP2J2 and sEH in human grade 2 colorectal carcinoma tissues and adjacent normal tissues.

Fenofibrate and WY-14643 affected p-p38 levels in both undifferentiated and differentiated cells of both cell lines. The PPARα inhibitor affected p-p38 levels only in undifferentiated HT-29 cells. Surprisingly, undifferentiated HT-29 cells showed an opposite response to PPARα activators treatment than the rest of the cells tested, namely, differentiated HT-29 as well as Caco2 cells regardless of their differentiation status. Fibrates increased p-p38 in undifferentiated HT-29 cells, whereas in other cases p-p38 expression was decreased.

It is known that p-p38 phosphorylates substrates both in the cytoplasm and in the nucleus. In the cytoplasm, p38 can phosphorylate other kinases such as MNK1/2, while in the nucleus it activates transcription factors such as ATF2, p53 and STAT1 [40]. HT-29 and Caco2 cell lines differed in the subcellular localisation of p-p38. HT-29 cells showed p-p38 predominantly in the cytoplasm and PPARα ligands affected its expression in this cellular compartment, whereas Caco2 cells showed higher nuclear positivity and fibrates affected p-p38 expression in both compartments.

The different response of p-p38 expression to PPARα ligands could be influenced by the differentiation status of the cells. Although HT-29 and Caco2 cell lines are both derived from grade 2 colorectal adenocarcinomas, there are differences between them as they carry different mutations: HT-29 in p53 and BRAF, while Caco2 in APC, p53 and SMAD4 [41,42,43,44]. As expected, we detected an increase in villin expression in differentiated HT-29 and Caco2 cells compared to undifferentiated cells. Surprisingly, we even detected significantly higher villin expression in undifferentiated Caco2 than in differentiated HT-29. This observation may explain the same response to PPARα ligands treatment in differentiated HT-29 as well as in undifferentiated and differentiated Caco2 cells, in contrast to undifferentiated HT-29.

As mentioned above, EETs are generally cytoprotective molecules with proliferative and anti-apoptotic functions [12,13]. EETs levels are regulated by a balance between EETs synthesis by CYP epoxygenases and EETs hydrolysis by sEH. Several studies have demonstrated that higher levels of EETs lead to a decrease in the expression of p-p38 in kidney, liver and lung [33,34,35,36]. Inhibition of sEH has been shown to prevent cisplatin-induced renal apoptosis. In the presence of EETs, p-p38 levels were decreased [36]. In mice, inhibition of sEH led to a decrease in p-p38 expression in mouse liver and attenuated high-fat diet-induced hepatic steanosis [35]. In the lung, inhibition of sEH attenuates lung toxicity and lung fibrosis by inhibiting p38 [33,34]. Our results in intestinal cells are consistent with these studies. We detected changes in the expression of p38, CYP2J2, and sEH in both undifferentiated and differentiated HT-29 and Caco2 cell lines. When we compared undifferentiated and differentiated cells, the differentiation process was accompanied by an increase in p-p38 expression as well as changes in the CYP2J2/sEH ratio, suggesting a decrease in the concentration of EETs in differentiated cells of both cell lines tested. Furthermore, the differentiated HT-29 and undifferentiated Caco2 showed comparable levels of p-p38 and sEH. These findings, as well as villin expression, suggest that undifferentiated Caco2 cell lines are more similar to differentiated HT-29 than to undifferentiated ones and support the idea that the effect of fibrates administration on p-p38 expression is dependent on the differentiation status of the cell.

Although fibrates could affect p-p38 levels, it remains questionable whether this phenomenon is PPARα-dependent. It has been described that the PPARα activators fenofibrate and WY-14643 upregulated p38 phosphorylation in melanoma cells [29] and in a mouse brain endothelial cell line [30]. On the other hand, fenofibrate inhibited p38 phosphorylation in renal proximal tubular cells [31]. However, all these studies concluded that the observed effect was PPARα-independent, as GW6471 did not reverse the effect of PPARα activators. In our study, it appeared that changes in p-p38 expression might also be PPARα-independent, at least in undifferentiated HT-29 cells, where administration of the PPARα activators fenofibrate and WY-14643 led to the same result as the PPARα inhibitor GW6471. In more differentiated cells, GW6471 had no effect. However, to reveal the involvement of PPARα more clearly and directly, the co-treatment of the cells with PPARα inhibitor and PPARα activator would be beneficial.

The stage of tumour development may be an important determinant of the function of p38 in tumourigenesis. In general, low p38 activity impairs tumour formation and growth in early stages of the disease, while more advanced tumour stages may benefit from higher activation of the pathway [45]. In colorectal carcinoma, p38α protects intestinal epithelial cells from inflammation-induced colon cancer by regulating intestinal barrier function. However, p38α contributes to the proliferation and survival of colon cancer cells [46]. Deletion of p38 in the intestinal epithelium promotes colon tumourigenesis in mice [47].

To investigate whether EETs might be related to p-p38 levels in colorectal carcinoma, we examined tissue samples by immunohistochemistry. We examined only grade 2 colorectal carcinomas so in terms of degree of differentiation it is a homogeneous group of samples. We found significant differences in the subcellular localisation of p-p38 compared to normal adjacent colon tissue. In carcinomas, higher levels of p-p38 were detected in the cytoplasm, whereas in normal colonic surface epithelium p38 was nuclear positive. The previous study by Gulmann et al. described that the activated isoform of p38 was decreased in colorectal carcinoma compared to the corresponding normal mucosa [48], which is in good agreement with our results. The total level of p38 was unchanged in their study. The expression of p-p38 has been shown to be a negative independent prognostic factor for colorectal carcinoma. Overexpression of p-p38 predicted poor overall survival, disease-free survival, and distant metastasis-free survival [49]. In addition, p38α activation helps tumour cells survive chemotherapeutic treatments [45]. Subcellular localisation of p38 also appears to play a role. Inhibition of p38 nuclear translocation prevents colitis and colitis-associated colon cancer in mice [50]. From this point of view, a downregulation of p-p38 achieved by fibrates in differentiated HT-29 and undifferentiated as well as differentiated Caco2 should be desirable. However, careful further investigation is needed.

Although it has been reported that CYP2J2 expression is higher in colorectal carcinoma than in normal adjacent tissue [12], we found no difference in CYP2J2 expression in tumour tissue. This discrepancy could be explained by the fact that the previous study by Jiang et al. examined a very limited number of colorectal carcinoma samples (4 samples compared to 18 samples in our study). While the level of CYP2J2 was not altered in our tumour tissue samples, sEH expression was significantly lower in carcinomas, suggesting different concentrations of cytoprotective EETs. A higher concentration of EETs in carcinomas (as sEH expression is lowered) is associated with a decrease in p-p38 levels. This is consistent with our in vitro results and previous studies mentioned above [35,36], as well as our in vitro results.

In the current study, we used colorectal carcinoma cell lines derived from moderately differentiated carcinomas (grade 2) as well as patient tissue samples of the same grade. This approach enables consistent data to be obtained. According to our results, the effect of fibrates on p-p38 levels depends on the differentiation status of the intestinal cells. Thus, additional useful information could be obtained by using patient samples from well-differentiated (grade 1) and poorly-differentiated (grade 3) tumours.

In conclusion, fibrates increased p-p38 in undifferentiated HT-29 cells, whereas in other cases p-p38 expression was decreased. HT-29 cells showed p-p38 predominantly in the cytoplasm and PPARα ligands affected its expression in this cellular compartment, whereas Caco2 cells showed higher nuclear positivity and fibrates affected p-p38 expression in both compartments. Based on the expression of villin, p-p38 and sEH, it can be assumed that differentiated HT-29 and undifferentiated Caco2 cells share similar characteristics. Thus, the effect of fibrates may depend on the differentiation status of the cell. In colorectal carcinomas, higher levels of p-p38 were detected in the cytoplasm, whereas in normal colonic surface epithelium p-p38 showed nuclear positivity. The decrease in p-p38 positivity was associated with a decrease in sEH. Based on current knowledge, it can be concluded that fibrates affect the level of p-p38, but its exact role in the process of carcinogenesis remains unclear, so further research in this area is needed.

## 4. Material and Methods

### 4.1. Cell Culture and Treatment

The HT-29 and Caco2 cell lines are derived from human grade 2 colorectal carcinomas. Cells were cultured and treated under the same conditions and experimental scheme as described in our previous studies [51,52]. The cell lines were originally obtained from the American Type Culture Collection and authenticated by STR profiles prior to the experiment by the Department of Clinical Genetics, Palacky University Olomouc. Cells were routinely cultured in DMEM (Sigma Aldrich, St. Louis, MO, USA; D6171) supplemented with 10% (HT-29) and 15% (Caco2) FBS (HyClone, SV30160.03), penicillin (100 U/mL) and streptomycin (100 mg/L). Cells were incubated at 37 °C in 5% CO_2_ and passaged twice a week.

Cells were seeded onto 96-well culture plates (TPP, Cat. No. 92696) at a density of 10,000 cells/well (HT-29) and 7000 cells/well (Caco2) for In-Cell ELISA and onto 8-well cell culture slides (SPL Life Sciences, Pochon, Republic of Korea; Cat. No. 30108) at a density of 18,000 cells/well for immunocytochemical staining and allowed to adhere overnight. The cells were then treated with fenofibrate (Cayman Chemicals, Ann Arbor, MI, USA; Cat. No. 10005368), WY-14643 (Sigma-Aldrich, St. Louis, MO, USA; Cat. No. C7081) or GW6471 (Cayman Chemicals, Ann Arbor, MI, USA; Cat. No. 11697) as follows: 25 μM and 150 μM (HT-29) or 200 μM (Caco2) fenofibrate, 25 μM and 200 μM WY-14643, and 1 μM and 10 μM GW6471. Cells were incubated at 37 °C for 72 h. Stock solutions of PPARα ligands were prepared by dissolving in DMSO. Control cells were treated with corresponding concentrations of DMSO. The concentrations used were determined using the WST-1 proliferation assay (Roche, Basel, Switzerland; Cat. No. 11 644 807 001). The results of this assay under the same experimental conditions were published in our previous studies [51,52].

Differentiated HT-29 cells were obtained by treatment with 5 mM sodium butyrate (Sigma Aldrich, St. Louis, MO, USA; B5887) for 72 h. Differentiated Caco2 cells were obtained by postconfluent growth for 14 days with growth medium changes twice a week [53]. After differentiation, the medium was changed and the cells were treated with fenofibrate, WY-14643, or GW6471 for 72 h as described above. Differentiated cells used as controls were treated with appropriate concentrations of DMSO instead of PPARα ligands. Cells were not reseeded during the experiments.

### 4.2. In-Cell ELISA (ICE)

Changes in the expression of proteins of interest were assessed using the In-Cell ELISA colourimetric kit (ThermoScientific, Waltham, MA, USA; #62200). The procedure was performed according to the manufacturer’s protocol. After the incubation period, cells were washed in PBS and fixed with 4% paraformaldehyde for 10 min at RT. Prior to primary antibody incubation, cells were permeabilised with 0.01% Triton-X100 (included in the kit) and pretreated with 1% hydrogen peroxide (20 min, RT). The samples were incubated overnight at 4 °C with the following primary antibodies: phospho-p38 MAPK alpha (Thr180, Tyr182) (Invitrogen, MA5-15177) at 1:250 dilution, villin (GeneTex, Hsinchu, Taiwan; GTX110034) at 1:1500 dilution, CYP2J2 (Novus Biologicals, Engelwood, CO, USA; NBP2-46419) at 1:100 dilution, EPHX2 (GeneTex, GTX84570) at 1:50 dilution. The samples were then incubated with horseradish conjugate followed by TMB substrate (part of the In-Cell ELISA colourimetric kit). The measured antibody signals (absorbance at 450 nm) were normalised to whole cell staining using Janus Green (absorbance at 615 nm). Results are expressed as relative expression [%] compared to the corresponding control cells (100%). Absorbance was measured using a Power Wave XS microplate reader (Bio-Tek). The experiment was performed in four independent duplicates (*n* = 8).

### 4.3. Immunocytochemical Staining

After the incubation period with fenofibrate, WY-14643 and GW6471, the samples were fixed with 4% paraformaldehyde for 10 min at RT. Prior to staining, the slides were rehydrated, cell membranes were permeabilised with 0.01% Triton-X100 and heat-induced antigen retrieval was performed in EDTA pH9 (120 °C, 15 min, Histos apparatus). After pre-treatment with PolyDetector Peroxidase Blocker (Bio SB, part of the detection kit) for 5 min and ProteinBlock (Dako, Golstrup, Denmark) for 30 min, samples were incubated with primary antibody against phospho-p38 MAPK alpha (Thr180, Tyr182) (Invitrogen, Waltham, MA, USA; MA5-15177) at 1:75 dilution for 1 h at RT. Visualisation was performed using the Mouse/Rabbit PolyDetector DAB HRP Brown Kit (Bio SB, Santa Barbara, CA, USA; Cat. No. BSB 0205). Tris buffer with TWEEN 20 (pH 7.6) was used for washing between different steps. Cell nuclei were counterstained with hematoxylin. The intensity of immunostaining was evaluated separately in the cytoplasm and in the nucleus. The scoring was semiquantitative as follows: 0 for negative tissue, 1 for weak signal, 2 for moderate signal, and 3 for strong signal. The evaluation was performed in 5 different fields of vision at 200× magnification and the percentage of cells in each category was counted. Finally, the histoscore for each treatment was counted as: H = [(1 × %weakly positive cells) + (2 × %moderately positive cells) + (3 × %strongly positive cells)]. Thus, the maximal value of 300 could be reached.

### 4.4. Immunohistochemical Staining

Basic patient characteristics of tissue samples from moderately differentiated (grade 2) colorectal adenocarcinoma and adjacent normal colonic tissue are shown in Table 1. The samples were obtained from the archives of the Department of Clinical and Molecular Pathology, Faculty of Medicine and Dentistry, Palacky University, Olomouc. The use of all samples was approved by the Ethics Committee of the University Hospital and the Faculty of Medicine and Dentistry of Palacky University in Olomouc (protocol no. 134/14 dated 21 August 2014). None of the patients received any anticancer treatment prior to surgery.

Antigens of interest were detected in 4 µm thick paraffin sections using the following primary antibodies: phospho-p38 MAPK alpha (Thr180, Tyr182) (Invitrogen, MA5-15177) at 1:75 dilution, CYP2J2 (Novus Biologicals, NBP2-46419) at 1:50 dilution, and sEH (Santa Cruz, sc166916) at 1:200 dilution. After deparaffinisation and hydration, heat-induced antigen retrieval was performed in citrate buffer pH6 (CYP2J2 and sEH) or EDTA pH9 (p38) (120 °C, 15 min, Histos device). The samples were then pretreated with PolyDetector Peroxidase Blocker (Bio SB, part of the detection kit) for 5 min and with ProteinBlock (Dako) for 30 min. Slides were incubated with primary antibodies for 1 h at RT and the reaction was visualised using the Mouse/Rabbit PolyDetector DAB HRP Brown Kit (Bio SB, Cat. No. BSB 0205). Tris buffer with TWEEN 20 (pH 7.6) was used for washing between different steps. Cell nuclei were counterstained with hematoxylin. Slides were then washed in tap water, dehydrated, and coverslipped.

Staining intensity was scored semiquantitatively as follows: 0 for negative tissue, 1 for weak signal, 2 for moderate signal, and 3 for strong signal. Due to the subcellular localisation of the studied antigens, the staining of CYP2J2 and sEH was evaluated in the cytoplasm, whereas the staining of p38 was evaluated in the nucleus and cytoplasm separately. To prevent observer bias, the slides were coded before scoring and the slides were scored twice at different times.

### 4.5. Statistics

The results obtained from the In-Cell ELISA were evaluated by one-sample *t*-test against the control value of 100%. The results of immunohistochemical staining of tissue samples were evaluated by the Wilcoxon signed rank test. All calculations were performed using Graph Pad Prism 8 at a significance level of *p* < 0.05. Statistically significant differences are marked with an asterisk (*) directly in the graphs: * *p* ≤ 0.05, ** *p* ≤ 0.01, *** *p* ≤ 0.001, **** *p* ≤ 0.0001.

## Figures and Tables

**Table 1:** Basic patients’ characteristics.

No.	Sex	Age	Diagnosis	Tumour Localization	TNM Staging			Grading
					T	N	M	
1	male	71	adenocarcinoma	colon sigmoideum	T3	N0	M0	G2
2	female	76	adenocarcinoma	colon descendens, rectum	T3	N0	M0	G2
3	male	72	adenocarcinoma	colon descendens, rectum	T2	N0	M0	G2
4	male	80	adenocarcinoma	colon sigmoideum	T3	N0	M0	G2
5	female	74	adenocarcinoma	colon descendens, rectum	T2	N0	M0	G2
6	male	76	adenocarcinoma	colon sigmoideum	T4a	N0	M0	G2
7	male	72	adenocarcinoma	colon sigmoideum	T3	N1a	M0	G2
8	male	52	adenocarcinoma	colon sigmoideum	T2	N0	Mx	G2
9	male	59	adenocarcinoma	colon sigmoideum	T3	N2a	M0	G2
10	male	77	adenocarcinoma	colon sigmoideum	T3	N1	Mx	G2
11	male	70	adenocarcinoma	colon sigmoideum	T4a	N0	M1a	G2
12	male	57	adenocarcinoma	colon sigmoideum	T2	Nx	Mx	G2
13	male	71	adenocarcinoma	colon sigmoideum	T2	N0	M0	G2
14	female	68	adenocarcinoma	colon sigmoideum	T2	N0	M0	G2
15	male	60	adenocarcinoma	colon sigmoideum	T4a	N2a	M0	G2
16	male	47	adenocarcinoma	colon sigmoideum	T3	N0	M0	G2
17	female	73	adenocarcinoma	colon descendens, rectum	T2	N0	M0	G2
18	male	55	adenocarcinoma	colon sigmoideum	T3	N2b	M0	G2

## Data Availability

Data is contained within the article.
